# Heart and Breathing Rate Variations as Biomarkers for Anxiety Detection

**DOI:** 10.3390/bioengineering9110711

**Published:** 2022-11-19

**Authors:** Florian Ritsert, Mohamed Elgendi, Valeria Galli, Carlo Menon

**Affiliations:** 1 Biomedical and Mobile Health Technology Lab, ETH Zurich, 8008 Zurich, Switzerland; 2 Department of Mechanical and Process Engineering, ETH Zurich, 8092 Zurich, Switzerland; 3 Menrva Research Group, Schools of Mechatronic Systems Engineering and Engineering Science, Simon Fraser University, Burnaby, BC V5A 1S6, Canada

**Keywords:** digital health, wearable technology, heart rate variability, respiration rate variability, breathing rate variability, anxiety assessment, mental health monitoring, real-time anxiety detection

## Abstract

With advances in portable and wearable devices, it should be possible to analyze and interpret the collected biosignals from those devices to tailor a psychological intervention to help patients. This study focuses on detecting anxiety by using a portable device that collects electrocardiogram (ECG) and respiration (RSP) signals. The feature extraction focused on heart-rate variability (HRV) and breathing-rate variability (BRV). We show that a significant change in these signals occurred between the non-anxiety-induced and anxiety-induced states. The HRV biomarkers were the mean heart rate (MHR; $\bar{p}$ = 0.04), the standard deviation of the heart rate (SD; $\bar{p}$ = 0.01), and the standard deviation of NN intervals (SDNN; $\bar{p}$ = 0.03) for ECG signals, and the mean breath rate (MBR; $\bar{p}$ = 0.002), the standard deviation of the breath rate (SD; $\bar{p}$ < 0.0001), the root mean square of successive differences (RMSSD; $\bar{p}$ < 0.0001) and SDNN ($\bar{p}$ < 0.0001) for RSP signals. This work extends the existing literature on the relationship between stress and HRV/BRV by being the first to introduce a transitional phase. It contributes to systematically processing mental and emotional impulse data in humans measured via ECG and RSP signals. On the basis of these identified biomarkers, artificial-intelligence or machine-learning algorithms, and rule-based classification, the automated biosignal-based psychological assessment of patients could be within reach. This creates a broad basis for detecting and evaluating psychological abnormalities in individuals upon which future psychological treatment methods could be built using portable and wearable devices.

## 1. Introduction

Stress and related psychological conditions, primarily anxiety and depression, are a significant societal burden, and nearly 300 million people are globally affected [1], with the prevalence increasing as of 1990 [2]. The COVID-19 pandemic globally exacerbated these effects [3]. On a psychological level, stress and related mental health diseases, particularly depression and anxiety disorders, are important topics not only in modern Western societies but worldwide. The COVID-19 pandemic has had a significant negative impact on average stress levels and mental health. In 2020, the American Psychological Association wrote, “We are facing a national mental health crisis that could yield serious health and social consequences for years to come” [4].

As a result of these circumstances, the need for stress-reducing measures is very high. The standard approach to assess anxiety is based on questionnaires (e.g., the Beck Anxiety Inventory and the Hamilton Anxiety Rating Scale) during a medical interview. With the increasing demand for mental-health assistance during the ongoing pandemic, there is often a substantial delay before patients can access medical attention. Another major drawback of the medical interview approach is the strong subjective component of the questionnaire responses. Therefore, there is a need for the more objective and continuous evaluation of a patient’s emotional status in order to prevent exacerbation of the effects and provide timely intervention. In addition to the increase in the use of wearable devices, monitoring electrocardiogram (ECG) signals or respiration (RSP) rate provides new possibilities to continuously monitor emotional states. One option could be to directly improve the brain status via wearable devices. However, to achieve that goal, a person’s psychological status must first be assessed. Our previous study showed that ECG and RSP signals are especially useful for detecting stress and anxiety with technical examination methods [5,6].

Several other studies employed ECG [7,8] and RSP signals [9,10] as physiological measures for an anxiety state. Identifying biomarkers for anxiety is an essential first step to developing a machine-learning algorithm for the automatic detection of anxiety [11]. Heart-rate variability (HRV) is one of the most prominent biomarkers when using ECG signals to investigate emotions, as outlined in a recent review [12]. Breathing-rate variability (BRV) was also extensively investigated [10,13]. Therefore, HRV and BRV biomarkers for anxiety detection are examined in this study.

## 2. Materials and Methods

### 2.1. Data Used

The Anxiety Dataset 2022 [14] was used, which contains 19 participants, 14 males and 5 females (age 26.15 ± 8.69 years; range of ages: 18–56 years) from different cultural backgrounds. All the participants were students recruited from Simon Fraser University in Canada. The data on ECG and RSP signals were obtained with a portable device (MP45, BIOPAC Systems, Inc., Goleta, CA, USA) and were recorded at a sampling frequency of 500 Hz. While the signals were being recorded, the participants were watching eight video clips of different lengths (between 1 and 15 min) that alternated between being anxiety-inducing and non-anxiety-inducing (see Table 1). The recorded data in units of mV were saved in a MATLAB (ver. 2020b) array for further processing. The dataset is publicly available in the Figshare repository https://figshare.com/articles/dataset/Anxiety_Dataset_2022/19875217, accessed on 22 September 2022.

A distinctive feature of the present study is that we did not use an emotionally “neutral” video; to advance the investigation, the video clips were split into those that had a nontransitional video phase in the center of the video clip and those that had a transitional video phase at the boundary with the assumption that a difference in HRV and BRV could be found within the nontransitional phase but not within the transitional video phase. This approach is based on the consideration that the change in the recorded physiological signals resulting from the induced anxiety state does not happen instantaneously or rather it is not as fast as the transition between the two types of videos (anxiety-inducing vs. non-anxiety-inducing). This assumption is based on the excitation transfer theory that postulates that, after removing the stimulus (here, switching from anxiety-inducing to non-anxiety-inducing videos), the individual would have residual arousal.

### 2.2. Data Preprocessing

To remove the typical noises of ECG and RSP signals, such as baseline wander, powerline interference, and electromyographic (EMG) and electrode motion artifact noise [15,16], the ECG and RSP signals were bandpass-filtered (Butterworth filter) in MATLAB with the following settings: ECG data were filtered with a second-order Butterworth filter with bandpass frequencies of 0.5 and 20 Hz, following the recommendations of Hejjel and Kellenyi [17]; RSP data were filtered with a second-order Butterworth filter with bandpass frequencies of 0.04 and 0.3 Hz, following the manufacturer’s instructions for the utilized sensor (belt) and the recommendations of Jhuang and Ma [18]. Next, the data were chronologically split into eight segments according to the eight video clips for data analysis.

### 2.3. Data Analysis

To analyze the filtered data, HRV/BRV analysis was conducted using Kubios HRV software (ver. 3.5.0, available at http://www.kubios.com, accessed on 22 September 2022). HRV/BRV refers to variations in both instantaneous heart/breathing rates and the intertime series between consecutive peaks. Therefore, we first extracted the peak-to-peak intervals for the ECG data (RR intervals) with the built-in function of Kubios HRV, and with the MATLAB tool breathTimes for RSP data (https://de.mathworks.com/matlabcentral/fileexchange/81066-breathtimes, accessed on 22 September 2022).

These steps were followed by the HRV/BRV analysis of ECG/RSP data in a duration range of our video clips that equated to short-term HRV/BRV analysis. As described by McNames and Aboy [19] (HRV), Castaldo et al. [20] (HRV), and Bandara and Wijesiriwardana [21] (BRV), the mean heart rate (MHR)/mean breathing rate (MBR), standard deviation (SD), root mean square of successive differences (RMSSD) of the R peaks, and standard deviation of the NN intervals (SDNN) are among the more popular features used to assess HRV and BRV. The NN interval is analogous to the RR interval, but refers to the normalized ECG data after removing an artifact [22]. The data were further sorted between clips with a nontransitional video phase in the center of the video and those with a transitional video phase at the boundary to test of our assumption that there would be a difference in the signals, with an effect not arising for the transitional video phases where confounding emotions are at play. Following the recommendations of McNames and Aboy [19], a segment length of 60 s was chosen for the nontransitional video phase in the center of the video, which was then analyzed using a segment by segment using Kubios. In this way, the indices were calculated for each 60-second segment. For the transitional phase, 15 s were chosen. The transitional phase was located at the beginning and end of each video clip.

### 2.4. Statistical Analysis

The statistical analysis was conducted with MATLAB (ver. R2020b). First, the indices were extracted from the MATLAB files exported from Kubios HRV in the previous step. Then, the indices of all the participants, which had been separated by the non-transitional video phase and the transitional video phase, were combined into one array for each video clip. The video clip arrays of each type of video anxiety-inducing and non-anxiety-inducing, were finally combined into four arrays, two for the non-transitional video phase and two for the transitional video phase. To investigate a statistically significant difference, ANOVA was used to test for a mean difference and the Wilcoxon test was used to determine the difference in the median. The average of both *p*-values was used as an overall evaluation of significance.

## 3. Results and Discussion

We evaluated the changes in the recorded ECG and RSP signals between the transitional and nontransitional phases, and between non-anxiety-inducing vs. anxiety-inducing video clips. Time domain features for the ECG and RSP signals, and frequency domain features for ECG signals were evaluated: features in the anxiety-inducing vs. non-anxiety-inducing clips were compared using the ANOVA and Wilcoxon tests. On the basis of a review of several studies, Castaldo et al. [20] described the expected change in the HRV indices when stress was induced. According to this meta-analysis, MHR should increase for stress-induced states, whereas the other time domain HRV indices (SD, RMSSD, SDNN) should decrease. RMSSD is a measure of interbeat interval variance. Our results agreed with these expectations with the exception of MHR, which was lower for the anxiety-inducing state. The same results should apply to RSP, since HRV and BRV are related [21].

**Figure 1:**
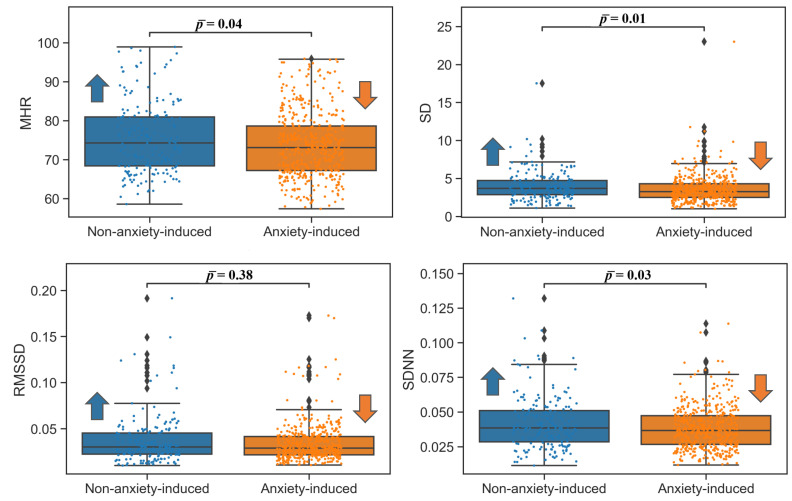
Box plots of the time domain ECG features in the nontransitional video phases. Mean heart rate: MHR (bpm); standard deviation of the heart rate: STDHR; root mean square of successive differences: RMSSD; standard deviation of NN intervals: SDNN. Arrows indicate which of the two populations (non-anxiety-induced state or anxiety-induced state) had a higher (upward arrow) or lower (downward) value for ease of visualization.

For the frequency domain, the low-frequency (LF) power (0.04–0.15 Hz) accounts for the activation of both parasympathetic and sympathetic systems, while the high-frequency (HF) power (0.15–0.4 Hz) is associated with parasympathetic system activation. A low LF/HF ratio reflects parasympathetic dominance, while a high LF/HF ratio indicates sympathetic dominance [22]. The results of this study agreed with these expectations, except for MHR, which decreased. The changes in the ECG time domain features in the nontransitional video phase showed lower MHR, SD, and SDNN results for the anxiety-inducing clips. For the RMSSD, no statistically significant difference was observed (p=0.18, ANOVA; p=0.58, Wilcoxon; $\bar{p}$ = 0.38). For the transitional video phase, no significant changes were found for any of the ECG time domain HRV measures. All the ECG time domain results with corresponding *p*-values are summarized in Table 2, and the corresponding box plots are shown in Figure 1.

**Figure 2:**
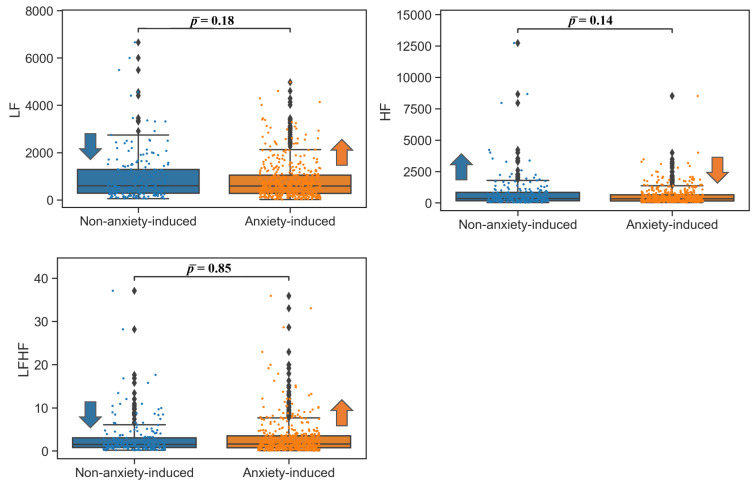
Box plots of frequency domain ECG features in nontransitional video phases. Low frequency: LF [ms${}^{2}$]; high frequency: HF [ms${}^{2}$]. Arrows indicate which of the two populations (non-anxiety-induced state or anxiety-induced state) had a higher (upward arrow) or lower (downward) value for ease of visualization.

**Figure 3:**
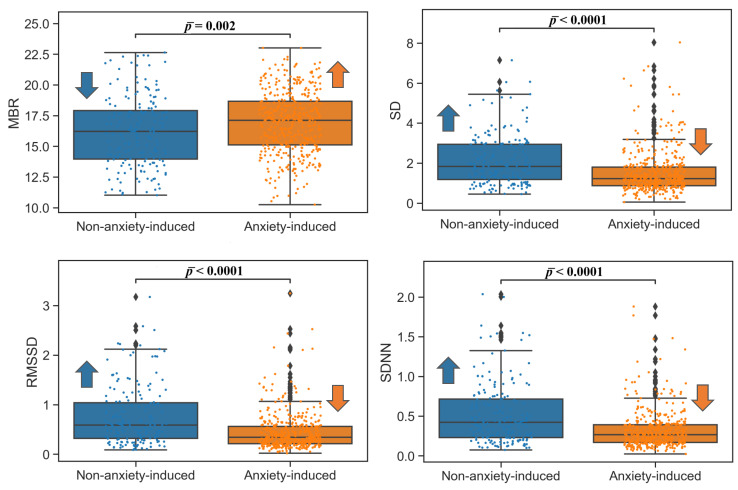
Time domain RSP box plots nontransitional video phases. Mean breathing rate: MBR (bpm); standard deviation of the breathing rate: STDBR; root mean square of successive difference: RMSSD; standard deviation of NN intervals: SDNN. Arrows indicate which of the two populations (non-anxiety-induced state or anxiety-induced state) had a higher (upward arrow) or lower (downward) value for ease of visualization.

For the ECG frequency domain, a significant increase in LF and a decrease in HF were observed, as was an increase in LF/HF ratio. The transitional video phase again yielded no significant difference between the anxiety-inducing and non-anxiety-inducing recordings. The results for the ECG frequency domain are summarized in Table 3, and the box plots are shown in Figure 2. The results for ECG were reinforced by the results for RSP: the BRV time domain indices (SD, RMSSD, SDNN) were lower in the nontransitional phase for the anxiety-inducing video clips in comparison to the non-anxiety-inducing video clips. A small increase (4.7%) in MBR was observed for the anxiety-inducing state in comparison to the lower MHR detected from the ECG signals (Table 2). All BRV analysis results were significant for the nontransitional video phase, and were not significant for the transitional video phase according to the ANOVA and Wilcoxon tests (Table 4 and Figure 3).

On the basis of a review of several studies, Castaldo et al. [20] described the expected change of HRV indices when stress was induced. According to this meta-analysis, MHR should increase for stress-induced states, whereas other time domain HRV indices (SD, RMSSD, SDNN) should decrease. In particular, SDNN at a resting state is strongly correlated to respiratory sinus arrhythmia (RSA) [23], which was decreased when inducing both positive and negative emotions [13]. The same pattern should apply to RSP, since HRV and BRV are related [21]. Moreover, RSP is closely related to the ANS activity: negative mood states such as anxiety and stress activate the sympathetic part of the ANS, and result in faster and shallower breathing. Our results for all indices agreed with these expectations except MHR, which was lower for the anxiety-inducing state. This is an interesting finding as it shows an inverse relationship between HR and HRV indices, which was reported in the literature [24].

For the frequency domain features of the ECG signal, the LF power (0.04–0.15 Hz) accounts for the activation of both parasympathetic and sympathetic systems, whereas the HF power (0.15–0.4 Hz) is associated with parasympathetic system activation and reflects the change in HR related to the respiratory cycle [22]. LF power is expected to increase in anxiety-induced states due to vagal activity when RSP rates are very low (below 8.5 resp/min) [22] and HF power is expected to decrease with negative emotions, such as anxiety, worrying, stress, and panic [12]. Thus, the LF/HF ratio reflects the induced shift of the ANS balance towards sympathetic activation and parasympathetic withdrawal; hence, it should also increase [25], although the LF/HF ratio is a more controversial feature as it was originally based on 24 h recordings [23].

RSP is closely related to ANS activity: negative mood states, such as anxiety and stress, activate the sympathetic part of the ANS, and result in faster and shallower breathing, as confirmed by our findings of an increase in MBR during stress induction. Overall, the effects of stress on the physiological state can be effectively measured with ECG and RSP signals, reflecting changes in ANS activity. Corresponding HRV/BRV analysis provides detailed information about cardiac and respiratory activity. In the time domain, in addition to the HRV and BRV, there was variation in the intertime series between consecutive peaks. In the frequency domain, LF/HF activity was examined, which is in direct relation to vagosympathetic balance. In the present study, stress was induced by means of video clips that were alternately anxiety-inducing and non-anxiety-inducing. The results of the study showed a significant decrease in the HRV and BRV time domain variability measures, specifically for SD (HRV, BRV), RMSSD (BRV), and SDNN (HRV, BRV), for the stress-induced states. This is in accordance with the majority of studies in the literature that investigated the influence of stress on HRV, as summarized in the meta-analysis by Castaldo et al. [20], and the influence on BRV, as discussed by Bandara and Wijesiriwardana [21]. In contrast to the literature, in the present study, MHR decreased (p=0.03, ANOVA; p=0.049, Wilcoxon; $\bar{p}$ = 0.04). MBR was significantly increased for the stress-induced states, again in accordance with previous findings [26].

## 4. Conclusions

In conclusion, this study proved the possibility of monitoring the state of anxiety induced through videos by assessing objective measures: HRV and BRV. The novelty of introducing a transitional phase between the stress-induced and non-stress-induced states, and the subsequent analysis shed new light on the findings in the currently available literature about the influence of stress on HRV and BRV. This new finding enables ECG and RSP signals to be interpreted in a recognizable and reproducible manner. This work provides a foundation for the further systematic processing of human mental–emotional states assessed via ECG and RSP signals. Identifying potential biomarkers is a prerequisite for establishing real-time data analysis and interpretation of future ECG and RSP signals collected via portable and wearable devices.

## Figures and Tables

**Table 1:** Video clip details: clip number, anxiety-inducing (AI)/non-anxiety-inducing (NAI), description, and duration.

Video Clip Details			
Clip Number	AI/NAI	Description	Duration
Clip 1	AI	Animation, puppy, amputation	3${}^{\prime }$16${}^{\prime }$${}^{\prime }$
Clip 2	NAI	Animation, music	1${}^{\prime }$59${}^{\prime }$${}^{\prime }$
Clip 3	AI	Animation, orphan	3${}^{\prime }$38${}^{\prime }$${}^{\prime }$
Clip 4	NAI	Happy clips and images	1${}^{\prime }$37${}^{\prime }$${}^{\prime }$
Clip 5	AI	Natural disasters	5${}^{\prime }$17${}^{\prime }$${}^{\prime }$
Clip 6	NAI	Minions	3${}^{\prime }$51${}^{\prime }$${}^{\prime }$
Clip 7	AI	Car crashes	14${}^{\prime }$42${}^{\prime }$${}^{\prime }$
Clip 8	NAI	Nature	7${}^{\prime }$00${}^{\prime }$${}^{\prime }$

**Table 2:** ECG time domain HRV features. Mean heart rate: MHR; standard deviation (SD) of heart rate; root mean square of successive differences: RMSSD; standard deviation of NN intervals: SDNN; non-anxiety-induced (NAI); anxiety-induced (AI).

ECG Time Domain HRV Features							
	Feature	Mean (NAI, AI)	SD (NAI, AI)	Range	p-Value ANOVA	p-Value Wilcoxon	$\bar{p}$-Value
Non-transitional	MHR	75.40, 73.75	8.93, 8.41	57.37–98.95	0.03	0.049	0.04
	SD	3.95, 3.55	1.88, 1.80	0.98–23.00	0.01	0.002	0.01
	RMSSD	0.03, 0.03	0.02, 0.02	0.01–0.19	0.18	0.58	0.38
	SDNN	0.04, 0.03	0.01, 0.01	0.01–0.13	0.01	0.05	0.03
Transitional	MHR	77.88, 76.62	9.46, 8.88	65.75–95.08	0.68	0.59	0.63
	SD	4.10, 4.30	1.70, 1.89	1.53–7.27	0.33	0.97	0.65
	RMSSD	0.03, 0.04	0.01, 0.02	0.01–0.09	0.22	0.45	0.34
	SDNN	0.06, 0.04	0.09, 0.01	0.01–0.46	0.41	0.97	0.69

**Table 3:** ECG frequency domain HRV features. Note: low-frequency: LF [ms${}^{2}$] (non-anxiety-induced/anxiety-induced); high-frequency: HF [ms${}^{2}$]; LF/HF ratio: LF/HF; non-anxiety-induced (NAI); anxiety-induced (AI).

ECG Frequency-Domain HRV Features							
	Feature	Mean (NAI, AI)	SD (NAI, AI)	Range	p-Value ANOVA	p-Value Wilcoxon	$\bar{p}$-Value
Non-transitional	LF	968.90, 823.39	1055.0, 802.79	14.85–6659.1	0.06	0.29	0.18
	HF	758.70, 545.88	1372.1, 696.71	22.70–12740	0.01	0.27	0.14
	LF/HF	2.93, 2.96	4.35, 4.07	0.06–35.92	0.92	0.79	0.85
Transitional	LF	790.11, 643.20	625.27, 546.89	57.35–2179.7	0.48	0.39	0.44
	HF	546.75, 734.15	338.76, 777.12	90.73–3113.7	0.38	0.85	0.62
	LF/HF	1.83, 1.35	1.68, 1.15	0.09–6.24	0.36	0.52	0.44

**Table 4:** RSP time domain BRV Features. Mean breath rate: MBR; standard deviation of breath rate (SD); root mean square of successive differences: RMSSD; standard deviation of NN intervals: SDNN; non-anxiety-induced (NAI); anxiety-induced (AI).

RSP Time-Domain BRV Features							
	Feature	Mean (NAI, AI)	SD (NAI, AI)	Range	p-Value ANOVA	p-Value Wilkoxon	$\bar{p}$-Value
Non-transitional	MBR	16.14, 16.90	2.87, 2.53	10.25–23.01	0.002	0.002	0.002
	SD	2.13, 1.52	1.28, 1.06	0.05–8.03	<0.0001	<0.0001	<0.0001
	RMSSD	0.76, 0.45	0.59, 0.38	0.01–3.24	<0.0001	<0.0001	<0.0001
	SDNN	0.52, 0.32	0.38, 0.23	0.02–2.03	<0.0001	<0.0001	<0.0001
Transitional	MBR	16.32, 16.84	2.71, 2.48	10.88–21.43	0.58	0.65	0.61
	SD	2.71, 1.94	1.42, 1.22	0.63–5.84	0.11	0.08	0.1
	RMSSD	0.79, 0.64	0.49, 0.55	0.13–2.02	0.43	0.13	0.28
	SDNN	0.55, 0.43	0.33, 0.32	0.12–1.25	0.31	0.09	0.2

## Data Availability

The Anxiety Dataset 2022 is publicly available and can be downloaded via this link: https://figshare.com/articles/dataset/Anxiety_Dataset_2022/19875217.
